# Liquid Biopsy in the Clinical Management of Cancers

**DOI:** 10.3390/ijms25168594

**Published:** 2024-08-06

**Authors:** Ho-Yin Ho, Kei-See (Kasey) Chung, Chau-Ming Kan, Sze-Chuen (Cesar) Wong

**Affiliations:** Department of Applied Biology & Chemical Technology, The Hong Kong Polytechnic University, Hong Kong SAR, China; hoyin.kenny.ho@gmail.com (H.-Y.H.); ckskasey@gmail.com (K.-S.C.); kantrevor@gmail.com (C.-M.K.)

**Keywords:** liquid biopsy, clinical management, cancers

## Abstract

Liquid biopsy, a noninvasive diagnosis that examines circulating tumor components in body fluids, is increasingly used in cancer management. An overview of relevant literature emphasizes the current state of liquid biopsy applications in cancer care. Biomarkers in liquid biopsy, particularly circulating tumor DNA (ctDNA), circulating tumor RNAs (ctRNA), circulating tumor cells (CTCs), extracellular vesicles (EVs), and other components, offer promising opportunities for early cancer diagnosis, treatment selection, monitoring, and disease assessment. The implementation of liquid biopsy in precision medicine has shown significant potential in various cancer types, including lung cancer, colorectal cancer, breast cancer, and prostate cancer. Advances in genomic and molecular technologies such as next-generation sequencing (NGS) and digital polymerase chain reaction (dPCR) have expanded the utility of liquid biopsy, enabling the detection of somatic variants and actionable genomic alterations in tumors. Liquid biopsy has also demonstrated utility in predicting treatment responses, monitoring minimal residual disease (MRD), and assessing tumor heterogeneity. Nevertheless, standardizing liquid biopsy techniques, interpreting results, and integrating them into the clinical routine remain as challenges. Despite these challenges, liquid biopsy has significant clinical implications in cancer management, offering a dynamic and noninvasive approach to understanding tumor biology and guiding personalized treatment strategies.

## 1. Introduction

**Figure 1:**
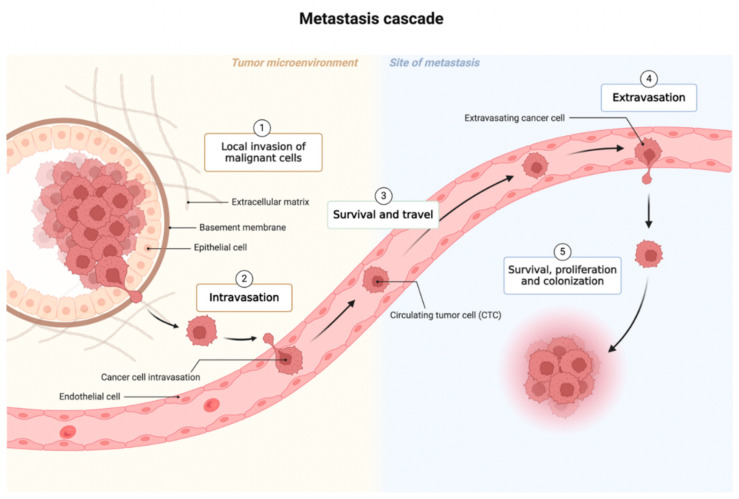
Metastasis cascade. This figure was created with BioRender (https://biorender.com) (accessed on 10 June 2024).

Cancer is the leading cause of death in the world, accounting for nearly 20 million new cancer cases and 9.7 million deaths in 2022. Cancer is also referred to as a silent killer since its symptoms are vague and thus difficult to detect early. Cancer formation consists of the following four phases: initiation, promotion, progression, and metastasis. The first phase, initiation, involves gene variants in a cell. Promotion is the phase between a premalignant lesion and the development of invasive cancer, involving the accumulation of actively proliferating preneoplastic cells. The next phase is progression where genetic and phenotypic changes and cell proliferation occur. At this phase, the tumor size increases rapidly and the cells may undergo further variations with invasive and metastatic potential [1]. The final stage is metastasis, which refers to the spread of cancer cells from a primary tumor to distant organs of the body. The metastasis process is a multistep process, named “metastatic cascade”, broadly divided into five distinct steps (Figure 1). The malignant cells invade into adjacent tissues and penetrate into lymphatic and circulatory systems. These malignant cells are now known as “CTCs”. These cells then exit from the lymphatic and circulatory systems into adjacent normal tissue or organs, which then survive and proliferate in adjacent tissue or organs, leading to colonization [2].

**Figure 2:**
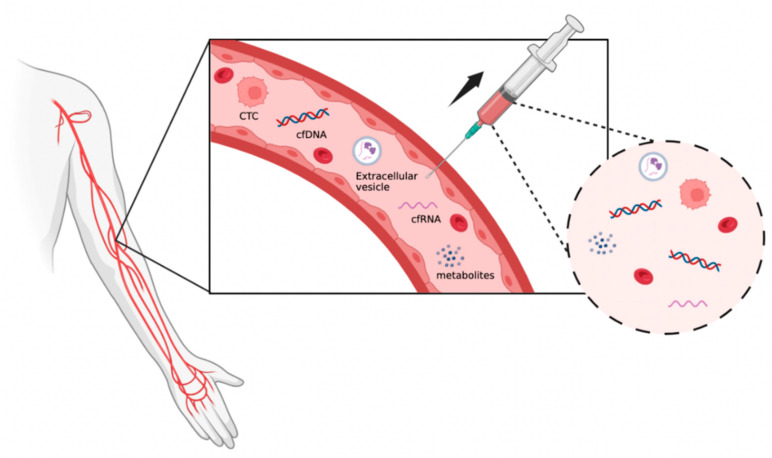
Biomarkers present in liquid biopsy. This figure was created with BioRender (https://biorender.com) (accessed on 10 June 2024).

Liquid biopsy has emerged as a revolutionary approach in the field of oncology, offering a minimally invasive and real-time method for the detection, monitoring, and characterization of cancer. Unlike traditional tissue biopsies, which are invasive and may not always capture the heterogeneity of tumors, liquid biopsy involves the analysis of biomarkers in biofluids such as blood, urine, saliva, sputum, stool, ascites, pleural effusion, seminal plasma, or cerebrospinal fluid [3]. This approach holds great potential for transforming cancer management through the provision of clinicians with valuable insight into tumor dynamics, treatment response, and disease progression. In recent years, the field of liquid biopsy has made rapid advancements driven through technological innovations and a deeper understanding of cancer biology. The ability to detect CTCs, ctDNA, ctRNA, EVs, and metabolites in liquid biopsy samples has opened new avenues for personalized medicine and precision oncology [2] (Figure 2). With the aid of liquid biopsy, clinicians may be able to formulate tailor-made treatment strategies for individual cancers based on their individual molecular profiles, resulting in more effective and targeted therapies.

This review aims to explore the role of liquid biopsy in the clinical management of cancer, highlighting its potential applications, challenges, and future directions. By consolidating the current knowledge and research findings in this rapidly developing field, we seek to provide a comprehensive overview of the impact of liquid biopsy on cancer diagnosis, prognosis, treatment selection, and monitoring.

## 2. Background

Traditional methods of cancer diagnosis and monitoring have primarily relied on tissue biopsies, which involve the surgical removal of a sample of the tumor for analysis [4]. While tissue biopsies remain the gold standard for cancer diagnosis, they are limited by several factors, including their invasive nature, the possibility of tumor seeding [5], sampling errors, and a small risk of morbidity associated with tissue biopsy procedures [6]. These limitations underscore the need for noninvasive and dynamic approaches that can provide a more comprehensive picture of the disease. In addition, tissue biopsies typically obtain a sample of only a part of the tumor, thus only encompassing a part of tumor heterogeneity, limiting the information obtained on the levels of genetic and epigenetic variability of a patient’s cancer [7]. Liquid biopsy offers advantages over traditional tissue biopsies, providing a minimally invasive, dynamic, and comprehensive molecular analysis of tumors, aiding in early diagnosis, prognostication, and treatment response monitoring (Table 1). By analyzing biomolecules shed by tumors into the bloodstream, liquid biopsy provides valuable information about the genetic alterations, variational profiles, and treatment response of cancer cells. Moreover, liquid biopsy can be performed after surgical resection and when there is no detectable metastatic mass for cancer treatment monitoring. Due to its minimally invasive nature, liquid biopsy can be performed multiple times or sequentially, which allows for continuous monitoring of disease progression as well as the emergence of treatment-resistant clones, offering insights that can be used to guide clinical decision-making. With the less invasive obtaining method and the various analytes that can be sampled in liquid biopsy, it can be used as a routine method for the real-time monitoring of cancer progression, early diagnosis of cancer, and assessing treatment response [8]. Liquid biopsy may help reveal intra-tumor heterogeneity (within tumors) and inter-tumor heterogeneity (between tumors), allowing the differences between the primary tumor and metastases, and the differences exposed during disease progression to be distinguished [9]. For instance, Jin et al. successfully identified three potential prognostic ctRNA biomarkers, which may be possible to apply MRD testing involving the identification of any remnants of cancer cells after operation [10]. Through the integration of liquid biopsy into clinical practice, cancer management will be revolutionized by early detection, personalized treatment selection, and real-time monitoring of treatment outcomes. Researchers are exploring liquid biopsy’s utility in diverse cancer types and clinical settings, where the field has great potential to improve patient outcomes and expand our understanding of cancer biology.

Although liquid biopsy holds several advantages over tissue biopsy, more studies and advanced technologies are required to be conducted and invented in order for liquid biopsy to have the potential to replace tissue biopsy. The analytes in liquid biopsy are present in a very low concentration, thus the assays for the detection of analytes should be sensitive and specific enough in order to produce a valid result for the detection and monitoring of cancer. Moreover, tumor histology specification and staging cannot be determined by liquid biopsy, stating that liquid biopsy should be conducted with tissue biopsy for the treatment and diagnosis of cancer. Studies have shown that validation is still needed to increase the potential of liquid biopsy [10,11]. Therefore, implementing liquid biopsy assays in clinical practice is indeed facing significant challenges despite the promising potential of this technology.

## 3. Technology to Collect and Detect Liquid Biopsy

**Figure 3:**
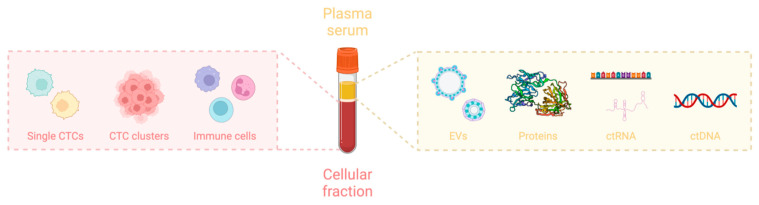
Biomarkers in blood. This figure was created with BioRender (https://biorender.com) (accessed on 10 June 2024).

In liquid biopsy, biomarkers are identified in biofluids in order to provide valuable insights into the presence and characteristics of cancer. The main components of liquid biopsy include CTCs, ctDNA, ctRNA, EVs (exosomes), and proteins as shown in Figure 3. These biomarkers shed by tumors into the bloodstream or other body fluids can offer valuable insights into the genetic changes and heterogeneity of cancer cells. Among different types of body fluids, blood is considered the most common material to analyze, but other body fluids may hold some benefits over blood. For instance, the malignant pleural and peritoneal effusions may exhibit a higher concentration of CTCs than blood, or collecting CTCs in urine can enable the diagnosis of bladder cancer without the need for endoscopy [12].

### 3.1. Circulating Tumor Cells (CTCs)

Cells that shed from a tumor and enter the circulatory or lymphatic system are referred to as CTCs. They are able to travel through the bloodstream or lymphatic system to other areas of the body, which has the potential to cause distant metastases. Different molecular markers are present on different CTCs depending on the type of cancer. As most of the cancers are of an epithelial origin, a “universal” epithelial marker of cancer, an epithelial cell adhesion molecule (*EpCAM*) can be used as a common marker for CTCs [13]. The expression of an *EpCAM* varies in different cancers with breast and prostate cancer being the highest and neurogenic cancers being the lowest. Cancer-specific CTC marks such as epidermal growth factor receptor-2 (*HER2*), prostate-specific membrane antigen (*PSMA*), estrogen receptor, folate receptor, and survivin are in accordance with the specific molecular markers of the primary tumor [14]. Since CTCs are found to have a very low concentration in the blood even in patients with metastatic cancer [15], highly sensitive technologies are required to detect and isolate these cells.

#### 3.1.1. Capture and Isolation of CTCs

##### Advanced Microfluidic Technologies

**Figure 4:**
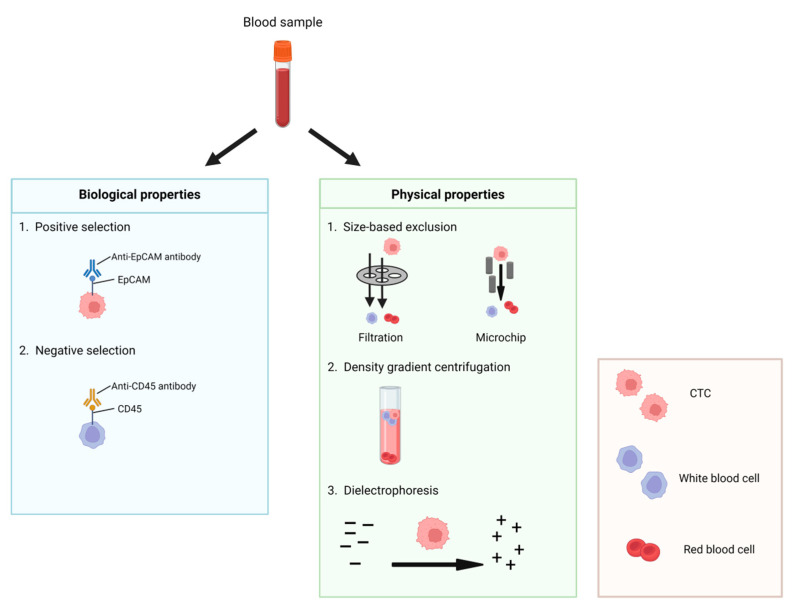
The isolation methods of CTCs. This figure was created with BioRender (https://biorender.com) (accessed on 9 June 2024).

Microfluidic devices offer high-throughput, high-resolution isolation and analysis of CTCs from blood samples. These platforms use microscale channels to capture and isolate CTCs based on physical properties such as size, density, deformability, or electric charges [16], or based on biological properties like the expression of specific protein markers, allowing for more sensitive and specific detection of rare CTCs (Figure 4).

In terms of biological properties, *EPCAM*-based isolation is considered a reliable method. However, in recent findings, CTCs can reduce the expression of specific markers, which allows them to escape from detection and result in false negative results. For instance, when CTCs undergo epithelial–mesenchymal transition (EMT), *EpCAM* expression is down-regulated while the expression of mesenchymal markers like *N-cadherin* increases [17,18]. In this case, negative selection can be adopted. Negative selection refers to the depletion of nonmalignant blood cells from blood using antibodies, such as targeting cell surface antigen CD45 on white blood cells [19]. It is noted that the drawbacks of negative selection include a lower purity of CTCs relative to the positive selection approach and the risk of depleting CTCs owing to being trapped in a mass of immune cells [20,21,22].

In terms of physical properties, CTCs show a larger size than normal blood cells. Size-based exclusion methods such as the isolation by size of epithelial tumor cells (ISET) filtration system have been developed to enable *EpCAM*-independent isolation of CTCs. The blood passes through the pores to allow size exclusion of CTCs so that larger CTCs are trapped [23]. However, this ISET filtration system has limitations including a lower purity of CTCs and the potential loss of smaller CTCs. Density gradient centrifugation is a fast separation method but it has a lower sensitivity. Dielectrophoresis (DEP) can separate CTCs from normal blood cells based on their unique dielectric properties. The dielectric properties depend on diameter, membrane area, density, conductivity, and volume, although they can be altered during prolonged storage [12].

##### Examples of Microfluidic Devices

The CTC-Chip can capture CTCs by coating the micro posts with different molecular markers such as anti-*EpCAM* antibodies. Using gold nanoparticles on a herringbone chip (HB-Chip) can isolate and release CTCs safely by chemical ligand-exchange reaction [14]. The CTC-iChip can undergo immunomagnetic sorting of CTCs by label-free, positive, and negative selection methods based on the antigens used [24]. All in all, microfluidic devices facilitate the efficient isolation of CTCs by different mechanisms.

#### 3.1.2. Strategies on CTCs Analysis

**Figure 5:**
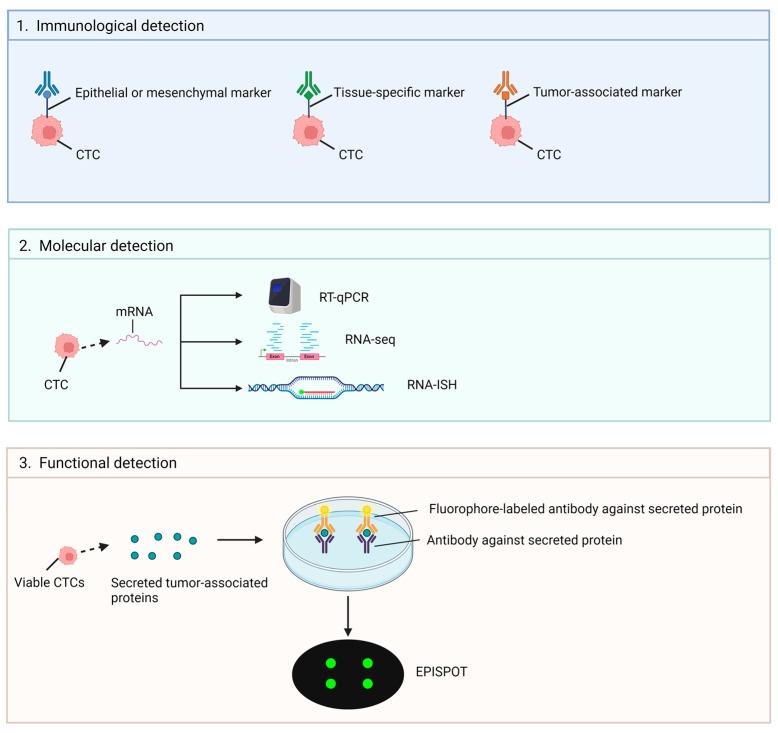
The detection methods of CTCs. This figure was created with BioRender (https://biorender.com) (accessed on 9 June 2024).

After the isolation of CTCs, they can be identified by immunological, molecular, or functional assays as shown in Figure 5 [25].

##### Immunological Technologies

In the immunological assay, CTCs are detected by antibodies against epithelial, mesenchymal, tissue-specific, or tumor-associated markers. For instance, not only *EpCAM* but also some of the epithelial markers including cytokeratin members and *E-cadherin* or mesenchymal markers like *vimentin* (*VIM*) and *fibronectin* can be detected [14]. Tissue-specific markers like prostate-specific antigen (*PSA*) show a high specificity to prostate cancer and tumor-associated markers like *HER2* are overexpressed in breast cancer. It is important to note that the population of CTCs shows heterogeneity, indicating that there is a wide variety of CTC markers among various cancer types, even in the same patient [14]. Therefore, it is challenging to define the origin of the CTC population by using limited molecular markers only.

#### 3.1.3. Molecular Technologies (RNA-Based)

Tumor cells have changed at genetic and transcriptomic levels, allowing them to escape from the primary tumor and survive as CTCs [26], hence RNA analysis of CTCs can be used for detection and quantification. CTCs are identified by quantitative reverse transcription polymerase chain reaction (RT-qPCR), RNA sequencing (RNA-seq), and RNA in situ hybridization (RNA-ISH). RT-qPCR is a frequently used technique to detect and monitor gene expression in CTCs due to its high sensitivity and cost-effectiveness when compared to other RNA-based detection methods [26]. Since the concentration of CTCs is often low in blood, droplet digital PCR (ddPCR) may also be applied. The CTCs’ RNA is separated into many partitions by lipid droplets to improve the detection of CTCs in the early stage of cancer due to the increased concentration of CTCs’ RNA and maximized amplification in a lipid droplet [27]. RNA-ISH allows the detection of localized RNA in CTCs without denaturing the cells. It can be used without any enrichment process to reduce the risk of losing CTCs during processing while obtaining a high sensitivity, namely the CTCscope method to detect eight epithelial markers and three EMT markers [28,29]. RNA-seq can study a large number of genes simultaneously in either total CTCs or a single CTC to determine the gene expression profile. Bulk RNA-seq demonstrates the average gene expression in a CTC population, while single-cell RNA-seq enables the analysis of cellular heterogeneity and identification of cellular types according to their genes in CTCs, thus illustrating the precision advantage of single-cell analysis over bulk analysis [30,31]. Although CTC transcriptomics can be used for detection and providing valuable insights about the phenotype of individual CTCs, the main challenge is the reduced stability of RNA when compared to DNA, thus a validated protocol is required to avoid any analytical errors and false results [32].

##### Single-Cell Analysis of CTCs Using Microfluidic Devices

A study conducted by Aceto et al. (2014) demonstrated a high level of concordance between expression patterns of CTC clusters and single CTCs, highlighting the importance of single-cell resolution RNA sequencing [33]. Single-cell analysis of CTC clusters provided insight into tumor cell migration and metastatic properties [34]. Huang et al. (2023) and Cheng et al. (2023) developed microfluidic devices for the isolation and analysis of CTCs, emphasizing the importance of efficient capture and release of CTCs in cancer diagnosis and monitoring [35,36]. Cheng et al. (2023) introduced a poly(ethylene oxide) concentration gradient-based microfluidic device for the isolation of CTCs, highlighting the importance of efficient capture and release of these cells for cancer diagnosis and management [37]. Huang et al. (2023) developed a conductive nanofibers-enhanced microfluidic device for the efficient capture and electrical stimulation-triggered rapid release of CTCs [38]. This novel approach not only enables the effective detection of CTCs but also offers a mechanism for rapid release, which is crucial for cancer diagnosis and monitoring.

##### Functional Assays

Functional assays, such as drug sensitivity testing on isolated CTCs or CTC-derived xenograft models, can help predict treatment response and guide personalized therapeutic strategies based on the drug sensitivity profile of CTCs. Capturing viable CTCs can allow downstream culturing of CTC cell lines that are derived from patients [37]. Some methods can maintain CTC viability and culture possibility, such as density gradient centrifugation [38] and microfluidic chips [20]. However, all the mentioned methods require extra steps to demonstrate the CTCs’ viability. Therefore, the Epithelial Immuno-SPOT (EPISPOT) assay is developed to directly assess CTC viability with fewer steps.

EPISPOT is a method to detect viable CTCs only based on the specific tumor-associated proteins that are secreted, shed, and released by CTCs using an adaption of enzyme-linked immunospot (ELISPOT) technology [39]. Cell culture is required to accumulate enough amount of released marker proteins because the immunospots are the protein fingerprint created by viable cells only and the dead cells that do not secrete a sufficient amount of marker proteins are not detected [12]. The CTCs are cultured on a nitrocellulose membrane coated with specific antibodies, which are used to capture the specific protein secreted by CTCs. After that, the fluorophore-labeled antibodies are added to bind to the secreted proteins and the signal can be visualized under a fluorescence microscope [40]. EPISPOT often combines with the leukocyte depletion negative enrichment step and it can identify CTCs without epithelial features such as *EpCAM*-negative CTCs [39]. EPISPOT has already been validated for prostate cancer [41], breast cancer [42], melanoma [42], head and neck squamous cell carcinoma [43], and colorectal cancer [39], thus illustrating the clinical values of the assay.

### 3.2. Circulating Nucleic Acids

Circulating nucleic acids, including ctDNA and ctRNA, are fragments of nucleic acids released into the bloodstream by cells, including tumor cells. The release of circulating nucleic acids into the human circulatory system can originate from six sources, including apoptosis, necrosis, NETosis, erythroblast enucleation, EV, and exogenous sources [44].

### 3.3. Circulating Tumor DNA (ctDNA)

ctDNA is a portion of cell-free DNA (cfDNA) that is released from tumor cells and enters the circulatory system. The presence of epigenetic or genetic alterations, including tumor-specific methylation markers, rearrangements, copy number variations, and somatic point variants, allows ctDNA to be distinguished from normal cfDNA fragments [45]. The genetic and epigenetic modifications of ctDNA molecules may reflect the genome or epigenome of the cell of origin, thus it can be used to estimate prognosis, for identification of residual disease and treatment selection, and/or indicate potential risk of relapse [46]. The half-life of ctDNA in the circulatory system varies from minutes to 2.5 h [47] and the levels of ctDNA are generally very low, which may require highly sensitive technologies for the detection of ctDNA.

### 3.4. Circulating Tumor RNA (ctRNA)

RNA released from cells through apoptosis, necrosis, and active secretion is referred to as cfRNA [10]. CtRNA, including microRNA (miRNA) and long non-coding RNA (lncRNA), are a portion of cfRNAs that are present in the body fluids of tumor patients. These cfRNAs can be isolated from various types of body fluids such as blood, urine, breast milk, and other fluids [48].

MiRNAs are small non-coding RNAs that are essential for the post-transcriptional regulation of gene expression. They are desirable biomarkers for noninvasive cancer diagnosis due to their stability in body fluids. Moreover, the expression patterns of miRNAs are tissue-specific, suggesting that miRNAs could be used as a biomarker for cancer diagnosis, prediction, and prognosis [49]. Numerous studies have shown that specific miRNA signatures are associated with various cancer types. miRNA-21 is an extensively studied miRNA that is found to be upregulated in a number of malignancies, including breast, lung, and colorectal cancer [50,51,52].

lncRNAs are transcripts with no protein-coding capacity. These RNAs act as regulatory factors that play an important role in different cellular processes such as cell growth, differentiation, proliferation, and apoptosis [48]. lncRNAs are bound to RNA-binding proteins and are released either inside apoptotic bodies or encapsulated in exosomes, making them resistant to RNase degradation. Thus, they can exist stably in the circulatory system and could be used as a noninvasive biomarker [53]. Studies have found that lncRNAs are expressed in a cell- and tissue-type specific pattern, allowing them to be used in the diagnosis of various cancers such as lung cancer, breast cancer, gastric cancer, liver cancer, and prostate cancer by measuring the expression level of lncRNAs [54].

#### 3.4.1. Isolation of Circulating Cell-Free DNA

After blood taking, it proceeds to plasma processing and DNA extraction. One of the main factors that affect the sensitivity of detecting ctDNA is the unintended release of cellular genomic DNA (gDNA) from the lysed blood cells [55]. Since ctDNA has a very low concentration in the peripheral blood, which already demonstrates a significant challenge to any analytical system, the unwanted increase in gDNA can further disturb the detection. Although it is discovered that a higher concentration of ctDNA is presented in the serum, the increased level may be caused by gDNA contamination due to the clotting process, resulting in diluting the targeted ctDNA concentration [56]. Hence, the plasma sample is a more reliable source of ctDNA to minimize the contamination from gDNA. The key to reducing the release of gDNA is to process the blood sample rapidly after collection without any delay and undergo a two-step centrifugation procedure for plasma preparation, which is recommended by many experts [57]. The two-step plasma preparation protocol includes a low-speed centrifugation (800–2000× *g*) to remove blood cells and prevent cell lysis, followed by a high-speed centrifugation (14,000–16,000× *g*) to remove cellular remnant to improve the plasma quality [58]. In view of the rigorous requirements such as rapid processing and the availability of high-speed centrifugation, an alternative approach of inhibiting nuclease activity in blood and stabilizing cells in the blood collection tube (BCT) can be adopted. The most common and cost-effective BCT is the EDTA collection tube, which can inactivate DNase activity. It is noted that the best performance is maintained when the EDTA tube samples are processed within 2 h after venipuncture [59], otherwise, there is a possibility of gDNA contamination from leukocytes when the blood storage time is more than 4 h [57]. Therefore, other commercial BCTs can be used when the plasma preparation time is more than 4 h, such as Streck Cell-Free DNA BCT [60], PAXgene Blood ccfDNA Tube [61], and Roche Cell-Free DNA Collection Tubes [62]. They can extend the processing time for at least 48–72 h, irrespective of temperature and without significant compromise to ctDNA detection [63]. 

In DNA extraction, there are different developed methods that can be classified into the following four methods: the magnetic bead–based method, the silica column–based method, the filtration-based method, and the phenol–chloroform-based method [64]. Comparing different types of commercial DNA extraction kits, they illustrate a large variation in terms of the extraction method, plasma input, throughput, and price [65]. The magnetic bead–based method has advantages regarding cost, speed, scalability, and automation, while the silica column–based method can obtain a higher yield [66]. Therefore, the choice of DNA extraction method is also an important factor to consider.

#### 3.4.2. Circulating Nucleic Acids Detection and Analysis

##### Digital PCR and Next-Generation Sequencing (NGS)

Ultrasensitive sequencing technologies are crucial for the analysis of ctDNA due to the low abundance of ctDNA in biological samples [67]. These technologies, such as ultradeep massively parallel sequencing with unique molecular identifier tagging enable the detection and quantification of minute amounts of ctDNA, which are typically released into the bloodstream by tumor cells undergoing cell death [67]. By utilizing ultradeep coverage sequencing methods, such as Illumina (San Diego, CA, USA) and Thermo Fisher (Waltham, MA, USA), researchers can achieve comparable performance to droplet digital PCR (ddPCR), like Bio-Rad (Hercules, CA, USA)’s QX200 and Thermo Fisher’s QuantStudio, for the detection and quantification of ctDNA from cancer patients [67,68]. For accurate identification of and quantification of genetic changes in ctDNA, the ultrasensitive nature of these sequencing technologies is essential, providing insight into tumor dynamics, treatment response, and disease progression based on the molecular profile of ctDNA, which ultimately guides personalized therapeutic strategies.

##### Methylation Profiling

Methylation analysis of ctDNA has been used in cancer diagnostics and monitoring. Numerous studies have investigated the importance of DNA methylation patterns in ctDNA for early detection, treatment assessment, and personalized medicine approaches. Tamkovich et al. (2022) discussed the potential use of aberrantly methylated DNA in liquid biopsy for patients with breast cancer, emphasizing its early involvement in cancer cell transformation [69]. A recent study by Markou and colleagues (2022) explored the role of DNA methylation analysis in early-stage non-small cell lung cancer (NSCLC) cases, demonstrating the potential for early cancer detection through DNA methylation analysis [70].

##### Fragmentomics Analysis

Fragmentomics analysis of cfDNA and ctDNA is essential for understanding the fragmentation properties of cfDNA, including the size distribution and biochemical characteristics of free ends. ctDNA is generally observed to have shorter fragment lengths compared to cfDNA [71]. Studies have shown that fragmentomic analysis provides comprehensive information about the entire cfDNA population, offering insights into tissue-of-origin and pathologies associated with altered fragmentomic profiles [72,73,74]. Moreover, cfDNA fragmentomics has been crucial in distinguishing cancer-derived cfDNA, determining the tissue of origin, and aiding in the early detection of cancer recurrence [75,76]. The analysis of cfDNA fragmentation patterns can also help infer the cell types contributing to the cfDNA milieu, such as cancer cells [77].

Additionally, cfDNA fragmentomic features have been shown to reliably discriminate between different disease states, highlighting the potential of liquid biopsy cfDNA fragmentomics in clinical diagnostics [78]. Furthermore, studies have emphasized the importance of understanding the origin and fragmentation mechanisms of cfDNA during physiological and pathological processes to leverage cfDNA as a molecular diagnostic tool [73]. By integrating cfDNA end motifs and fragment lengths, it is possible to identify active genes in liquid biopsies, providing insights into gene activity and underlying biological processes. Moreover, epigenetic profiling of cfDNA fragmentomes can also offer valuable information on DNA methylation patterns, aiding in predicting the CpG methylation of cfDNA without the need for bisulfite treatment [79]. Using fragmentomics to examine cfDNA fragments offers the potential for enhancing early cancer detection, monitoring treatment response, and developing personalized therapeutic strategies based on the molecular characteristics of cfDNA fragments.

### 3.5. Extracellular Vesicles (EVs)

EVs, including exosomes and microvesicles, ectosomes, oncosomes microparticles, and many others, are groups of membrane-closed vesicles containing DNA, RNA, proteins, and lipids enclosed in a phospholipid biolayer. They play a crucial role in intercellular communication and disease progression, including cancer [80]. In addition, EVs are secreted by all cell types and are present in various types of body fluids, allowing the collection of the biomarkers present in EVs for cancer diagnosis and management. For example, exosomes are small EVs that are emerging biomarkers for cancer diagnosis and guide treatments. Studies have found that exosomes are involved in the occurrence and development of various tumors, which act as carriers in tumor cells, allowing them to escape immune system surveillance [81]. Exosomes are also able to create a suitable microenvironment for tumor growth and guide the direction of tumor cell metastasis [82,83]. To improve the understanding of circulating EVs, researchers are integrating multi-omics approaches, implementing machine learning algorithms, and standardizing isolation and analysis protocols [84].

#### 3.5.1. EV Isolation and Characterization Technologies

The isolation and characterization of EVs are essential for understanding their functions and potential therapeutic applications. Several techniques have been developed and refined to address the challenges associated with EV isolation and characterization. The size exclusion chromatography (SEC) method is one of the most common methods for isolating EVs from plasma in recent years due to its ability to rapidly isolate relatively pure EVs [85,86]. Nevertheless, SEC is not suitable for all downstream applications, highlighting the need to develop a variety of isolation techniques to meet the needs of different research projects. An alternative method of EV isolation is ultracentrifugation but it can be time-consuming and difficult to implement in a clinical environment [87]. There have been recent advances in EV isolation efficiency and biomarker profiling by using membrane affinity–based methods and microfluidics [88,89]. These advancements are essential for improving diagnostic capabilities and advancing precision medicine initiatives. Furthermore, the development of novel techniques such as the EVtrap [90] and the salting-out approach [91] offer alternatives for achieving high purity and efficiency in EV isolation. Characterizing EVs is equally important and a variety of methods have been used for this purpose, including nanoparticle tracking analysis (NTA), transmission electron microscopy, and Western blotting.

##### EV Proteomic and RNA Profiling

The integration of proteomic and RNA profiling in EV research holds great potential for advancing precision medicine and improving disease diagnosis and monitoring. The development of EV proteomic profiling has been promising for the detection and monitoring of cancer, emphasizing the importance of understanding the protein cargo of EVs for clinical applications [92,93]. Through the use of NGS, comprehensive analyses of EV RNA content have been carried out, which have shed light on the multitude of RNA species present in EVs as well as the functional implications of their presence [94].

### 3.6. Metabolites

Metabolomics can be utilized to identify metabolites with a molecular weight of less than 1 kDa present in body fluids [95]. There has been increasing interest in metabolomic techniques in cancer research, highlighting their potential for identifying cancer-specific metabolites in liquid biopsy samples. For instance, metabolomics has been used to study dynamic responses and drug resistance in gastric cancer cells [96], analyze the metabolic changes associated with prostate cancer using integrated RNA and metabolite profiling of urine liquid biopsies [97], and develop metabolite classifiers for NSCLC diagnosis using ultrahigh-performance liquid chromatography-mass spectrometry/mass spectrometry (UPLC-MS/MS) [98]. In addition, Wei et al. (2021) utilized UPLC-MS to analyze serum metabolic features of gastric cardia adenocarcinoma (GCA) patients and healthy controls in order to identify a novel metabolic biomarker for early detection and prognosis in GCA patients [99]. Moreover, a study has demonstrated that benzoic acid might have the potential to be a diagnostic marker of colorectal cancer [100] and significant differences in metabolite levels can be found in different stages of the same cancer type [101]. The findings of these studies highlight the potential of metabolomics for advancing precision medicine and improving patient outcomes in cancer diagnosis, biomarker discovery, and treatment strategies.

## 4. Applications in Cancer Management

In cancer management, liquid biopsy plays a crucial role in several aspects. Liquid biopsy has demonstrated significant promise in early detection, diagnosis, monitoring cancer progression, treatment selection, and personalized medicine, as well as in detecting MRD and predicting relapse across different stages of cancer [102].

### 4.1. Early Detection and Diagnosis

Regarding early detection, liquid biopsy has transformed cancer screening by facilitating the identification of biomarkers in body fluids, enabling the detection of cancer at its early stages [103,104,105,106]. Due to the low amounts of ctDNA released by early tumors, research shows that variants in ctDNA alone have limited sensitivity for early-stage cancer detection. Consequently, different strategies were developed to improve sensitivity. For instance, a strategy called CancerSEEK, which involves the evaluation of ctDNA and circulating proteins such as carcinoembryonic antigen (CEA), cancer antigen 125 (CA-125), and cancer antigen 19-9 (CA19-9), has been proposed to increase the sensitivity for early detection [104]. Studies have demonstrated varying sensitivities across different cancer types, with the highest sensitivity for ovarian cancer and the lowest for breast cancer [8,50,107,108,109,110,111]. Therefore, combining ctDNA analysis with the assessment of circulating proteins can enhance the overall sensitivity for early detection.

In addition, ctRNA has shown promise in providing noninvasive information about gene expression without the need for biopsies through the use of circulating RNA sequencing [112]. Shen et al. (2023) emphasizes the importance of ctRNA in the early detection of cancer, emphasizing the clinical application of liquid biopsy in endometrial carcinoma [113]. In addition, miRNA is the most sufficient cfRNA molecule in body fluids so it also has the potential for enabling early diagnosis. For instance, various types of miRNA in sputum such as miR-145, miR-126, and miR-7 have been shown to obtain a high sensitivity and specificity to detect NSCLC [114]. However, there is a lack of consensus on miRNA panels to detect cancer development so validation studies of the discovered miRNA [115] and new studies of undiscovered miRNAs based on the genetic background of each population, type, and stage of cancer development is recommended [116].

Moreover, recent advances in liquid biopsy techniques, including the detection of exosome microRNAs and protein biomarkers, have made them more useful for diagnosing and monitoring cancer, providing real-time feedback and noninvasive disease assessment [103,117]. Recent advances in nanotechnology-enabled biosensors have allowed the detection of exosomal RNAs and proteins for cancer screening, diagnosis, and prognosis [118]. There has been evidence that short-length exosomal DNA or RNA derived from tumor cells can be detected more readily in liquid biopsy, demonstrating its high sensitivity [119]. Nanotechnology and antibody-conjugated signals in nanocavities have also been proposed to detect cancer markers in liquid biopsy [120].

### 4.2. Prognostication and Predictive Biomarkers

Recent studies have underscored the significant role of liquid biopsy in identifying prognostic and predictive biomarkers across various types of cancer, including esophageal cancer [121], prostate cancer [122], bladder cancer [123], colorectal cancer [10], and NSCLC [124]. Liquid biopsy has been shown to have both prognostic and predictive value in cancer management, offering molecular and phenotypic information about cancers [125]. The analysis of nucleic acids in liquid biopsies has demonstrated prognostic potential and predictive value for guiding treatment decisions in colorectal cancer and hepatocellular carcinoma [126,127].

### 4.3. Detection of Minimal Residual Disease (MRD)

Liquid biopsy has demonstrated significant promise in the detection of minimal residual disease (MRD) in various types of cancer. For instance, highly sensitive liquid biopsy assays have been successfully used to detect and characterize MRD in colorectal cancer patients. MRD occurs when persistent cancer cells spread from the primary lesion to distant organs or into the bloodstream when no radiological relapse has occurred after resection of the tumor [128]. In lung cancer, Sardarabadi et al. (2021) emphasized the use of liquid biopsy–based biosensors for detecting and assessing early stages of disease, including resistance to treatment and tumor mutational burden [129]. Furthermore, liquid biopsy is being used as a unique minimally invasive tool for breast cancer, diagnosing its MRD, studying its metastasis biology, and exploring its drug resistance mechanisms [130]. Moreover, it has been shown that ctDNA is useful in identifying MRD in locoregional gastric cancer, predicting recurrence, and guiding treatment regimens in the presence of MRD [131].

### 4.4. Treatment Selection and Personalized Medicine

The use of liquid biopsy to identify specific biomarkers in body fluids has significantly influenced treatment selection and personalized medicine. Several recent studies have highlighted the importance of liquid biopsy when selecting targeted therapies and evaluating treatment responses. Personalized medicine has benefited tremendously from liquid biopsy, enabling the identification of tumor-specific biomarkers that are used to optimize treatment regimens and monitor therapeutic outcomes [132,133]. The use of liquid biopsy has enabled accurate prediction, monitoring, and rational selection of appropriate therapies for individual patients by analyzing ctDNA and CTCs as well [134,135]. Aside from this, liquid biopsy has also been demonstrated to have promise in guiding therapeutic decisions for many types of cancer by providing real-time molecular information that is useful in guiding treatment choices and evaluating response to treatment. Lung cancer, breast cancer, colorectal cancer, and prostate cancer are introduced in this review because they were the global top four cancer types, respectively, in both sexes in 2022 in terms of incidence and mortality [136].

### 4.5. Lung Cancer

Lung cancer is one of the most common cancers with the highest mortality rate but with a low rate of early detection so most patients are diagnosed at the advanced stage, which leads to a lower survival rate. A prescreening program is thus an important tool to enable early detection and liquid biopsy can detect lung cancer in a safer and earlier way. Although tissue biopsy is regarded as the current gold-standard method, it may not be the best choice for detection or monitoring lung cancer. Firstly, a tissue biopsy may not be completely feasible because of the tumor location or performance status [137]. Secondly, tumor heterogeneity can result in noncomprehensive genomic profiles by tissue biopsy obtained in one site only [138]. Lung biopsy shows a high incidence of major and minor complications and tissue biopsy may yield insufficient DNA for genetic analysis [139]. Therefore, with the properties of noninvasiveness, simple accessibility, and great repeatability, liquid biopsy is considered a promising alternative to traditional tissue biopsy.

Lung cancer can be primarily classified into two types—NSCLC and small cell lung cancer (SCLC)—in which NSCLC accounts for about 85% of all cancer types [138]. A targeted therapy is developed for NSCLC patients to increase overall survival, namely the *EGFR* tyrosine kinase inhibitors (TKI) for patients with the *EGFR* variant. Kolesar et al. explored the use of liquid biopsy in guiding the selection of TKI therapies at various stages of lung cancer treatment to enhance the precision and efficacy of therapy for lung cancer patients with *EGFR* variants and resistance to standard treatments [140]. *EGFR T790M* is an acquired drug-resistant *EGFR*-dependent variant. Although SensiScreen EGFR Liquid assays are developed to detect *EGFR* variants at high sensitivity and specificity by using liquid biopsies as samples [141], *EGFR T790M* does not necessarily have to be screened for because of the development of Osimertinib, an approved treatment for *EGFR*-positive patients exhibiting a T790M resistance variant [142]. For other *EGFR*-independent variants that result in drug resistance, including Kirsten rat sarcoma (*KRAS*), phosphatidylinositol 4,5-bisphosphate 3-kinase catalytic subunit alpha (*PIK3CA*), *HER2*, RAF murine sarcoma viral oncogene homolog B1 (*BRAF*), and mesenchymal–epithelial transition (*MET*), they can be detected by liquid biopsy [143]. Hence, by integrating liquid biopsy with pharmacogenomics testing, lung cancer precision therapy can be enhanced by detecting tumors, monitoring variants and acquired resistance, and assisting in the selection of precise drugs through serial molecular profiling based on blood samples [140].

### 4.6. Breast Cancer

In breast cancer, there is an increase in both ctDNA and CTCs that correlate with cancer development. The analysis of these biomarkers is beneficial to manage advanced or early breast cancer patients. It is found that a high concentration of ctDNA is related to a more aggressive or relapsing disease [144]. CTCs are also useful in characterizing phenotypes and determining the possibility of metastatic spread and prognosis [145]. Hence, liquid biopsy shows its potential applications in diagnosis, treatment, and management.

Breast cancer can be classified into four subtypes based on molecular information, including luminal A, luminal B, *HER2*-positive, and triple-negative subtypes [146]. This molecular classification can categorize the patients who can benefit from the targeted therapy. However, when breast cancer becomes metastatic, it is difficult to be controlled. After confirming the status of hormone receptors and *HER2* by a biopsy of metastatic breast cancer lesions, the determination of metastatic phenotype can be performed by repeated sampling, which can provide useful information on molecular changes and treatment customization [147]. For example, the CD47 and PD-L1 markers in CTCs correlate with disease progression [148]. Moreover, the high rates of pSTAT3^+^ and TLR4^+^ in CTCs are detected in early-stage and metastatic patients, respectively [149]. The metabolic classification of CTCs has the potential to help recognize aggressive CTC subpopulations and discover new targeted therapies.

The detection of the *PIK3CA* variant is important for breast cancer because the *PIK3CA* variant can result in resistance to *HER2*-targeted therapies [150]. It is noted that there is a high incidence of discrepancy in the *PIK3CA* variant between primary breast cancer tumors and metastases, hence the detection of metastatic lesions is essential [151]. However, sufficient tumor tissue samples may not always be available or accessible for detection, or the tissue biopsy obtained at the time of surgical resection may not be able to reflect the current tumor molecular characteristics [151]. In view of that, liquid biopsy provides a real-time examination of genetic alterations in a minimally invasive way. In the SOLAR-1 trial, the assessment of *PIK3CA* mutational status was compared between formalin-fixed paraffin-embedded (FFPE) samples from the primary or metastatic sites, and ctDNA from the plasma in the same patient [152]. The result showed that *PIK3CA* variant testing on liquid biopsy achieves a higher specificity (97%) but lower sensitivity (55%) when compared to tissue biopsy. Similarly, a study by Nakai et al. also demonstrated that the *PIK3CA* variant is detected with a lower sensitivity in liquid biopsy, although heterogeneity related to the *PIK3CA* variant can be detected [153]. According to the results, the Therascreen PIK3CA RGQ PCR Kit (QIAGEN GmbH, QIAGEN Strasse 1, 40,724 Hilden, Germany) is approved by the U.S. Food and Drug Administration (FDA) to conduct the *PIK3CA* mutational analysis on both tissue biopsy by using gDNA from FFPE samples, and liquid biopsy by using ctDNA extracted from plasma samples [151]. The kit can detect 11 variants in *PIK3CA*, including exon 7 (C420R), exon 9 (E542K, E545A, E545D, E545G, E545K, Q546E, and Q546R), and exon 20 (H1047L, H1047R, and H1047Y) [154]. Therefore, liquid biopsy can provide valuable information for treatment selection and monitoring in breast cancer patients.

The estrogen receptor 1 (*ESR1*) variant plays a significant role in endocrine therapy (ET) resistance for metastatic breast cancer, and it can be detected by liquid biopsy. ddPCR and NGS are two commonly used methods for identifying *ESR1* variants in ctDNA, in which ddPCR provides higher sensitivity and accuracy, while NGS can evaluate larger regions like gene panels without prior knowledge of the variants [155]. Moreover, a study by Urso et al. assessed the concordance of the *ESR1* variant in tumor tissue samples from a metastatic lesion and ctDNA from plasma. The result demonstrates a high concordance rate between the tumor tissue and ctDNA (91%), indicating the reliability and feasibility of liquid biopsy for the ESR1 variant detection [156]. Compared to tissue sequencing, ctDNA may be superior because it can accurately reveal the molecular profile of metastatic cancer [152]. Therefore, integrating liquid biopsies into the diagnostic workflow in breast cancer patients can help highlight the resistance to ET and guide treatment decisions.

### 4.7. Colorectal Cancer (CRC)

In CRC, liquid biopsy has shown promise in various applications, such as early-stage detection, guiding and monitoring treatments, predicting relapses and metastases, revealing tumor heterogeneity, and detecting MRD [157].

In the early diagnosis and screening of CRC, hemoccult test and fecal immunochemical test (FIT) are two noninvasive detection methods that are mostly studied, but they demonstrate some limitations such as having a low sensitivity [158,159]. Hence, liquid biopsy was evaluated as an alternative and has shown satisfactory performance. CTCs have the potential to become a screening tool for early-stage CRC. Tsai et al. indicated a high sensitivity and adequate specificity of the CellMax (CMx) blood-based CTC test [160]. In addition, a combined analysis of the CTC count with an immunochemical fecal occult blood test (iFOBT) or serum Carcinoembryonic antigen (CEA) assay was conducted by Tsai et al., showing that the implementation of CTC testing can reduce the false positive rate of iFOBT and increase the disease detection rate of a serum CEA assay [161].

Apart from CTCs, ctDNA is also useful for CRC screening. Epigenetic changes including DNA methylation and histone modifications are some of the early events of carcinogenesis, which can be reflected in ctDNA to aid early diagnosis [162]. The FDA-approved Epi proColon test uses the real-time PCR assay to detect the methylation of septin9 (*SEPT9*) DNA in CRC patients [163]. However, it shows a limitation of having low sensitivity (68.2%) and specificity (79.1%) across all stages of CRC detection [164,165]. Another test, namely the ColoSure test, is a single-marker test to identify the methylation of *VIM* in CRC from stool DNA samples with a sensitivity ranging from 38% to 88% [166]. Recently, the ColoSure test has been replaced by the more efficient FDA-approved Cologuard test. It is a multitarget stool DNA test that assesses *KRAS* variants, methylation of bone morphogenetic protein 3 (*BMP3*) and Neuregulin 4 (*NRG4*), and hemoglobin concentration [167]. The sensitivity and specificity are reported to be 92.3% and 86.6%, respectively, showing the improved diagnostic accuracy of CRC [168].

Liquid biopsy can also provide insights for treatment monitoring and efficacy evaluation [169]. Boysen et al. investigated post-treatment ctDNA analysis in ddPCR and mass spectrometric–based multiplexed platform, MassARRAY (MA), showing that ctDNA can be used as a marker for MRD in the post-treatment of CRC [170]. Another study by Zhou et al. demonstrated the potential applications of ctDNA in locally advanced rectal cancer (LARC), such as reflecting real-time tumor bulk, predicting tumor response to neoadjuvant chemoradiotherapy (nCRT), and predicting the prognosis [171].

### 4.8. Prostate Cancer

The treatment of prostate cancer involves androgen deprivation therapy (ADT), which is regarded as the standard treatment and has shown its effectiveness in the initial management of advanced or metastatic prostate cancer. However, some patients develop resistance to the therapy, resulting in castration-resistant prostate cancer (CRPC) [172]. Androgen receptor signaling inhibitor (ARSI) is a drug blocking the androgen receptor at various levels that can prolong the survival of CRPC patients [173], but some patients also develop resistance to ARSI due to alterations in increasing AR transcriptional activity and target gene expression [174]. It is noted that the *PSA* marker, bone scintigraphy, computed tomography (CT), and positron emission tomography (PET) scans do not provide information on disease drivers or resistance mechanisms [175]. Other methods are required to provide a more comprehensive disease characterization, thus liquid biopsy assays are developed to provide insights into drug resistance mechanisms.

The androgen receptor splice variant 7 (*AR-V7*) is the most commonly detected variant associated with ARSI resistance and it can be used as a predictive variant to ARSIs such as enzalutamide and abiraterone acetate [176]. It can evade the action of anti-androgen therapies that target the ligand-binding domain (LBD) by retaining its DNA-binding domain but lacking the LBD [177], making it a negative treatment response. *AR-V7* detection can be achieved in CTCs and ctRNA, such as using the EPIC Sciences CTC AR-V7 nuclear localization assay [178] or the AdnaTest ProstrateCancerDetect AR-V7 quantitative polymerase chain reaction (qPCR) assay by QIAGEN [179]. According to a study by Nimir et al., CTC-based detection obtains a higher sensitivity and specificity for *AR-V7* when compared to ctRNA, indicating that CTC testing may be more reliable [180].

Homologous recombination repair (HRR) is responsible for repairing the double-stranded DNA breaks or interstrand cross-links. Defective DNA repair (homologous recombination deficiency, HRD) is regarded as a hallmark of prostate cancer [181]. *BRCA* (*BRCA1* and *BRCA2*) is a tumor suppressor gene that is critical for HRR by recruiting proteins such as RAD51 [182]. *BRCA* variants are consistently associated with an HRD phenotype in multiple cancers including prostate cancer, while other HRR pathway genes such as *ATM*, *PALB2*, or *RAD51* are also associated with an HRD phenotype [183]. It is observed that HRD can cause tumors to be more sensitive to poly(ADP-ribose) polymerase inhibitors (PARPi) treatment [183] such as olaparib or rucaparib. FoundationOne Liquid CDx test is an FDA-approved next-generation sequencing–based test to identify variants in *BRCA1*, *BRCA2*, and *ATM* in cfDNA to guide the treatment of olaparib or rucaparib [184]. Other tests, including BRACAnalysis CDx, Illumina TruSight Oncology 500 (TSO500), and Guardant360 CDx, are also developed by using plasma cfDNA as samples [185,186,187]. All in all, liquid biopsy offers a minimally invasive approach for detecting treatment resistance and variants to improve the guidance of treatment to patients.

CTC enumeration, such as the CellSearch assay, which is approved by the FDA [188], can be used for monitoring treatment response and prognosis. A study by de Kruijff et al. demonstrated that CTC count is independently associated with poor progression–free survival and overall survival of metastatic CRPC patients treated with cabazitaxel [189]. Apart from the CTC count, several markers in CTCs such as *PSMA* [190] or EMT markers [191] are also found to be associated with the prognosis of metastatic prostate cancer patients. Therefore, liquid biopsy may explore a better way for the assessment of prognosis.

## 5. Example of Clinical Trials Evaluating Liquid Biopsy for Cancer

There have been numerous clinical trials on liquid biopsy, including the detection of actionable variants, evaluating treatment response, and predicting prognosis. For instance, an ongoing clinical trial at the Jewish General Hospital in Montreal, Canada, called “TRICIA” (TRIPLE Negative Breast Cancer Markers In Liquid Biopsies Using Artificial Intelligence), is developing a test or score based on ctDNA expression within a cohort of patients with triple-negative breast cancer, which demonstrates the potential of liquid biopsy to guide treatment decisions based on specific breast cancer subtypes. [192]. Another prospective phase II clinical trial in CRC utilized liquid biopsies to detect spatial and temporal heterogeneity of resistance to anti-EGFR monoclonal antibodies by combining sequential profiling of ctDNA with mathematical modeling and imaging of tumor progression. This clinical trial showed that liquid biopsy can be used to monitor the progression of cancer and the response to treatment [193]. Furthermore, *EGFR* variant status is being evaluated in NSCLC patients using the ddPCR method in ongoing clinical trials, suggesting that liquid biopsy can play a major role in guiding treatment decisions for NSCLC patients [194]. As a result of these trials, it is evident that liquid biopsy can be useful for identifying specific variants in lung cancer patients and guiding targeted therapies.

## 6. Liquid Biopsy Regulatory Considerations and Challenges

Integrating liquid biopsy into clinical practice presents several regulatory challenges and considerations. Regulatory issues, standardization of methods, diagnostic performance, and further research are key factors affecting the integration of liquid biopsy in the clinic [195]. Challenges such as a lack of consensus in preanalytical and analytical processes, clinical validation, regulatory approval, and low amounts of CTCs, ctRNA, and ctDNA hinder the successful implementation of liquid biopsy in clinical settings. The success of regulatory pathways for liquid biopsy diagnostics has been attributed in part to the incremental value of FDA approval for tests developed under the Clinical Laboratory Improvement Amendment (CLIA), as well as their complexity, which can hinder their widespread replication in CLIA laboratories [196]. In addition to its clinical utility, liquid biopsy is used to noninvasively monitor tumor genomes and to ensure appropriate treatment for patients. However, liquid biopsy remains a challenge in effective cancer management due to the lack of sensitive and specific biomarkers, the limitations of sampling single primary tumors, and the difficulty of monitoring cancer progression over time [197].

Other the challenges faced in integrating liquid biopsy into clinical practice is the need to standardize techniques, validate assays, and interpret results [198]. Considering the complexity of liquid biopsy approaches, robust validation processes are required to ensure accurate and reliable results [199]. Moreover, tumor heterogeneity, clonality, and the dynamic nature of tumors pose challenges when interpreting liquid biopsy results for effective cancer management [200]. The lack of standardization leads to different issues such as low reproducibility of results, technical and biological variability, and difficulty in interpreting data, resulting in problems of integrating liquid biopsies into clinical practice [198].

To enhance the acceptance of liquid biopsy procedures in routine clinical practice, several strategies can be implemented based on the available literature. Firstly, the development and adherence to standardized protocols and guidelines are necessary. The implementation of liquid biopsy in clinical practice relies greatly on international standards, such as from the International Organization for Standardization (ISO). It is essential for different laboratories and settings to follow standardized protocols for sample collection, processing, and analysis. In cancer management, following international standards helps harmonize practices, improve data quality, and facilitate information exchange [201]. For example, the workflow and quality evaluation of sequencing data from massively parallel sequencing should be based on ISO20397-1:2021 [198] and ISO20397-2:2021 [198]. To be used in clinical laboratories, assays should follow ISO15189 [198]. A standardized liquid biopsy method and workflow could further enhance the clinical usefulness of this approach in cancer management. Healthcare providers can ensure accurate, reproducible, and clinically relevant liquid biopsy results by following established guidelines and protocols [162]. Additionally, standardizing approaches facilitate the sharing of data, collaboration, and integration of liquid biopsy into routine clinical practice [198,202]. Secondly, the validation of the assay according to the international standards is very important. For instance, the implementation of NGS-based genomic profiling assays using blood samples for analyzing cfDNA can overcome practical challenges with sample integrity and failure, enabling liquid biopsy in advanced solid tumor patients to be used clinically [203]. Thirdly, addressing preanalytical considerations such as standardizing collection methods, optimizing storage conditions, and ensuring proper handling of samples are essential to mitigate variability and improve the diagnostic accuracy of liquid biopsy procedures, which can assist in overcoming challenges related to low circulating tumor content and interpreting sequencing data, making liquid biopsy more feasible in routine clinical practice [204].

To ensure the accessibility and affordability of liquid biopsy for all patients, establishing standardized reimbursement policies and guidelines is crucial to help address cost barriers and ensure that all patients have equal opportunities to benefit from liquid biopsy technologies, ultimately improving cancer care and patient outcomes. In addition, the field of liquid biopsy can progress to provide more cost-effective cancer diagnosis and monitoring by creating user-friendly tools and promoting research and technological advancements. Moreover, healthcare systems can facilitate collaborations among researchers, industry partners, and regulatory bodies to drive innovation and cost reduction [205].

In the future, technological advances could significantly enhance the possibilities and applications of liquid biopsy in cancer diagnosis and treatment. Advances in technologies such as dPCR and NGS will revolutionize liquid biopsy analysis, making it possible to detect ctDNA/ctRNA and other biomarkers more precisely and sensitively [145]. Furthermore, the integration of microfluidics and artificial intelligence are expected to further improve the accuracy and efficiency of liquid biopsy, enabling real-time monitoring of disease progression and treatment responses [206]. As a result of these technological innovations, the utility of liquid biopsy is poised to increase in precision oncology, providing noninvasive and dynamic insights into tumor biology, treatment response, and disease monitoring [201]. The development of liquid biopsy will play an increasingly important role in personalized medicine, transforming cancer care and improving patient outcomes.

## 7. Conclusions

Liquid biopsy has emerged as an important tool in cancer management, providing noninvasive and real-time insights into tumor biology and guiding personalized treatment plans. Through the dynamic insight provided by liquid biopsy into tumor characteristics and treatment responses, liquid biopsy has profound clinical implications, revolutionizing personalized treatments and cancer diagnostics. While liquid biopsy has its challenges, such as standardization and cost-effectiveness, it holds enormous promise for improving cancer care and patient outcomes.

## Figures and Tables

**Table 1:** The comparison between liquid biopsy and tissue biopsy.

Liquid Biopsy	Tissue Biopsy
Samples derived from body fluid	Samples derived from needle biopsy or surgical biopsy
Minimally invasive	Invasive
Preserved by preservation reagents	Preserved by paraffin embedding or cryopreservation
Can be performed after surgical resection and when there is no detectable metastatic mass	Impossible to be performed after surgical resection and when there is no detectable metastatic mass
Does not allow tumor histotype specification and staging	Allows histological diagnosis and staging
Easily performed at multiple or sequential time points during treatment	Not suitable for frequent monitoring
Allows real-time monitoring of disease	Single information point over time and space
Potential to reflect tumor heterogeneity	Not suitable to reflect tumor heterogeneity
